# Supplementary material for: New technology for an ancient fish: A lamprey life cycle modeling tool with an R Shiny application
Source: PLoS One. 2025 May 14;20(5):e0323408. doi: 10.1371/journal.pone.0323408 (PMC12077736; doi:10.1371/journal.pone.0323408)
Supplement: Attachment — Submitted filename: PONE-D-24-5476_review.docx [file pone.0323408.s001.docx]

Overall comments

A well written paper describing a useful modeling tool. Two general comments and a few line-by-line edits

The GSA analysis – a plain language description of why this analysis was done would prepare the reader for considering the implications in the discussion. The MSE response variable throws me off as the response of interest for a sensitivity analysis. More detail on what this variable is comparing would be helpful. With a sensitivity analysis, I think of a measure of how much the response variable of interest changes (in this case the size of the spawner population) as we vary a parameter. I have a hard time placing your analysis in this conceptual framework. Not to say that it needs to be, but currently this section is hard for me to follow (both the methods and the rationale for why it is important).

Applications to Great Lakes sea lamprey control – this is mentioned in the first sentence of the discussion and in the conclusion, but the idea does not get developed at all in the text. If this is an important part of this work, the idea should be developed more in the text. An example of how it could be used or description of the management need that could be addressed would be helpful here. Similarities and differences to other DSTs that have been used to model sea lamprey dynamics in the Great Lakes would also be relevant to this discussion. As a starting point, Jones et al. (2009) and subsequent applications of this model would be relevant to discuss.

Jones, M.L., Irwin, B.J., Hansen, G.J.A., Dawson, H.A., Treble, A.J., Liu, W., Dai, W., Bence, J.R., 2009. An Operating Model for the Integrated Pest Management of Great Lakes Sea Lampreys. TOFISHSJ 2, 59–73. <https://doi.org/10.2174/1874401X00902010059>

Line by line comments

Figure 2 Caption.

Between and within year descriptions – from the model description,

Line 330 – burn in of 50 years in Fig. 3, doesn’t match description in text (few years)

Figure 3 Captions- I find the last sentence of the caption confusing/don’t follow the rationale. In cases where carrying capacity is based on data or even expert opinion for a particular stream, seems like the number of spawners is relevant model output.

Lines 494 – mean squared error response – what is being compared to what here? I usually think of using MSE to compare an estimated value to a known value. I get lost in the description of how it is applied here, I am not familiar with using MSE as a metric to describe a sensitivity analysis. Seems like a crucial concept to understand the significance of the whole GSA section and it gets lost on me. Some added plain language text on the rationale for the methods and why the analysis was done would be helpful.

Lines 612 – modeling local populations also become important when modeling the combined outcome form multiple stream-specific conditions/management actions. This allows for more realistic simulations.

Lines 697 –Is the point here that there will tend to be considerable uncertainty in life history parameters, particularly carrying capacity terms, so one shouldn’t put much weight on the absolute numbers of spawners as this is an equilibrium-type model (gets spun up with a burn-in period, rather than initialized at a specific value)? This could be stated more plainly. Also, if this is the case, why should we consider comparisons among management scenarios (relative number of spawners) as reliable but absolute numbers as unreliable? Digging into this question a bit more would help the reader here.

Lines 730 – an example of how it could help control efforts in the Great Lakes would be useful. This is brought up in the first line of the discussion as well, but never gets fleshed out with any detail.
